# Factors Influencing the Production Efficiency of Cloned Pigs: A Large-Scale Retrospective Analysis

**DOI:** 10.3390/ani16020168

**Published:** 2026-01-07

**Authors:** Huaxing Zhao, Shouquan Zhang, Xiaopeng Tang, Rong Zhou, Ranbiao Mai, Lvhua Luo, Qiaoyun Su, Sixiu Huang, Zhenfang Wu, Zicong Li, Gengyuan Cai, Junsong Shi

**Affiliations:** 1 National Engineering Research Center for Breeding Swine Industry, State Key Laboratory of Swine and Poultry Breeding Industry, National and Local Joint Engineering Research Center for Livestock and Poultry Breeding Industry, Guangdong Provincial Key Lab of Agro-Animal Genomics and Molecular Breeding, Guangdong Provincial Laboratory of Lingnan Modern Agricultural Science and Technology, Gene Bank of Guangdong Local Livestock and Poultry, College of Animal Science, South China Agricultural University, Guangzhou 510642, China; 2 Yunfu Branch, Guangdong Laboratory for Lingnan Modern Agriculture, Yunfu 527300, China

**Keywords:** pig, cloning efficiency, breeds, season, embryos transferred number

## Abstract

Pig somatic cell nuclear transfer (SCNT) has valuable applications in agriculture, biomedicine, and life sciences, yet low cloning efficiency remains a major constraint limiting its application. To systematically investigate factors related to the production efficiency of pig cloning, this study conducted a retrospective analysis of 367,701 SCNT embryos transferred into 2019 surrogate sows over five years, focusing on breeds of donor cells, the season of embryo transfers, and the number of embryos transferred per surrogate. Our data demonstrate that the genetic background of donor cells is a critical determinant. SCNT embryos generated by wild-type (WT) Pietrain and Duroc pigs yielded significantly higher cloning efficiencies compared to those from Large White and Yorkshire pigs. This breed-specific influence was also observed with genetically modified (GM) donor cells. Nevertheless, within the GM groups, GM-Duroc and GM-Yorkshire showed superior efficiency compared to GM-Large White and GM-Bama. Furthermore, Summer was identified as the least favorable season for embryo transfer, with significantly lower pregnancy rates, delivery rates, and cloning efficiency compared to the other seasons. Importantly, we established that transferring 100–150 embryos per recipient optimized cloning efficiency, significantly outperforming groups receiving higher embryo numbers without compromising pregnancy rates, delivery rates, or average litter sizes. Our findings provide valuable guidance for optimizing large-scale SCNT protocols in swine.

## Simple Summary

Pig cloning is an important technology, but its low efficiency limits wider application. By analyzing five years of data involving over 2000 surrogate sows and 367,000 embryos, we identified several important factors affecting cloning success. We found that the breed of the donor cell significantly influences outcomes, with notable differences observed between breeds. Compared to Summer, the other three seasons are more suitable for embryo transfer. Interestingly, using fewer embryos (100–150 per surrogate) resulted in better efficiency than using larger numbers. This contrasts with recommendations from a decade ago, suggesting that optimal embryo numbers need continual adjustment as cloning techniques improve. Our findings provide clear, practical guidance to improve the production of cloned pigs for agriculture and biomedical research.

## 1. Introduction

Somatic cell nuclear transfer (SCNT), or cloning, in pigs has valuable applications in agriculture [1], biomedicine [2,3], and life sciences [4,5]. Since the first batch of SCNT pigs was generated in 2000 [6], this technique has been extended to generate a population of elaborately selected boars with superior production performance [7,8] as well as genetically modified (GM) pigs. As recently as ten years ago, the efficiency of developing SCNT pig embryos to birth was only about 1% [9,10].

Animal cloning involves injecting a single donor cell into an enucleated mature oocyte, generating reconstructed embryos after electro-fusion, and transferring them into the uterus of the recipient animal. It is evident that the efficiency of animal cloning is influenced by factors related not only to oocytes, donor cells, embryo, and recipient animals, but also to other procedural and environmental variables. As previously reported, several efforts have been undertaken to improve porcine cloning, including the optimization of oocyte quality [11,12], donor cell preparation [13,14], the adjustment of fusion and activation [15,16], embryo transfer protocol [17], and the ovulation status of the recipient [17,18]. These studies have illustrated that optimizing the SCNT and embryo transfer procedures is necessary to improve the porcine cloning efficiency. Moreover, the number of embryos transferred is an important factor for porcine cloning efficiency. Although sows normally need only a minimum of four normal embryos to maintain pregnancy [19], because of the poor quality of SCNT embryos, people have generally transferred a large number of SCNT embryos to maintain pregnancy. With optimization procedures, the quality of SCNT embryos has been improved; thus, the number of embryos transferred needs to be re-evaluated.

Therefore, we conducted this retrospective study using data from cloned pig production in our laboratory to provide new insights for the pig SCNT field. In the present study, we investigated the influence of multiple factors on porcine cloning efficiency. We analyzed large-scale data from five years of cloned pig production, encompassing the transfer of 367,701 SCNT embryos into 2019 recipients, which resulted in 7781 cloned pigs. This dataset allowed us to evaluate the impact of season, donor cell breed, and the number of embryos transferred on key outcomes. The goal was to establish a more efficient procedure for large-scale production of pig cloning by evaluating the impact of the variable factors on pregnancy, delivery, and cloning efficiency.

## 2. Materials and Methods

### 2.1. Animals

All animal experiments in this study were approved by the Institutional Animal Care and Use Committee, South China Agricultural University. All efforts were made to minimize animal suffering.

### 2.2. Study Design and Data Collection

This retrospective study analyzed data from the large-scale production of cloned pigs at our facility from November 2021 to July 2025. Seasons for analysis were defined as follows: Spring (March–May), Summer (June–August), Autumn (September–November), and Winter (December–February). All embryo transfer procedures were conducted in Guangdong Province, China.

### 2.3. Oocyte Collection and Maturation

The ovaries of gilts were obtained from a local slaughterhouse and transported to the laboratory in normal saline with the antibiotics penicillin-G (100 IU/mL) and streptomycin sulfate (100 mg/L) at 30–35 °C within 5 h. Using a 10 mL disposable syringe with an 18-gauge needle, follicular fluid with cumulus–oocyte complexes (COCs) was aspirated from antral follicles (3–6 mm diameter), transferred to a 50 mL centrifuge tube, and allowed to stand for 20 min at 38.5 °C to aspirate the supernatant. Porcine COCs with at least three layers of compact cumulus cells and homogenous cytoplasm visible under a stereomicroscope were selected for in vitro maturation (IVM). The IVM medium is reported in our previous publication [20]. Approximately 50 COCs were transferred to each well of a four-well Nunc dish (Thermo Fisher Scientific, Waltham, MA, USA) into 500 μL of fresh IVM medium, and cultured in a humidified atmosphere at 38.5 °C with 5% CO_2_ for 44 h. Then, the surrounding cumulus cells were removed by treating the COCs with Dulbecco’s phosphate buffered saline (DPBS; Thermo Fisher Scientific, Waltham, MA, USA) containing 1 mg/mL hyaluronidase (Sigma-Aldrich, St. Louis, MO, USA) and 0.1% polyvinyl alcohol (Sigma-Aldrich, St. Louis, MO, USA) for 5 min at 38.5 °C by gentle pipetting for approximately 200 times. The oocytes were observed under a stereomicroscope, and those with the first polar body in the perivitelline space and intact cell membranes were selected for further cloning. Oocyte maturation culture was performed by the same core team following an identical protocol.

### 2.4. Preparation of Donor Cells

Donor cells were prepared as previously described [21,22]. Briefly, WT donor cells were obtained from an adult boar by ear biopsy. Ear tissues were immediately immersed in 75% ethanol, washed thrice with DPBS containing penicillin-G (100 IU/mL) and streptomycin sulfate (100 mg/L), and macerated into 1–2 mm pieces in Dulbecco’s Modified Eagle’s Medium (DMEM; Thermo Fisher Scientific, Waltham, MA, USA) supplemented with 10% fetal bovine serum (FBS; Thermo Fisher Scientific, Waltham, MA, USA). Then, fragments of ear tissue were seeded into the cell culture dishes and cultured in a humidified incubator with 5% CO_2_ at 37 °C; the culture medium was changed every other day. Fibroblast cells were harvested using 0.25% trypsin–EDTA (Waltham, MA, USA) and frozen in liquid nitrogen with the freezing medium (50% FBS, 40% DMEM, and 10% dimethyl sulfoxide). Primary fetus fibroblasts were isolated from 35-day-old male fetuses of Duroc, Large White, Yorkshire, and Bama pigs. Primary fetus fibroblasts were cultured in DMEM containing 12% FBS and 100 IU/mL penicillin-G/streptomycin in a humidified incubator with 5% CO_2_ at 39 °C. The transgene was mixed with a transposase, pCMV-hyPBase, and transfected into primary fetus fibroblasts by electroporation (BTX, San Diego, CA, USA). Then, the transfected cells were cultured in fresh culture medium. After 24 h, 300 μg/mL G418 (Gibco, Waltham, MA, USA) was added to the culture medium to select transfected cell colonies for about 15 days. The surviving cell colonies with EGFP expression were isolated within colony cylinders (Bellco Glass, Vineland, NJ, USA) and propagated in a fresh 24-well plate. Four colonies that proliferated well, with bright fluorescence, were then expanded and screened for the presence of the positive transgene.

Before SCNT, the fibroblasts were thawed and cultured in DMEM containing 10% FBS for 2–3 days until they reached 80–90% confluence. Adherent cells were harvested with 0.05% trypsin for 1 min and used for generating SCNT embryos.

### 2.5. Preparation of Somatic Cell Nuclear Transfer Embryos

Matured oocytes and a small number of donor cells were mixed in M199 medium (Gibco, Waltham, MA, USA) containing 2% FBS and 7.5 μg/mL cytochalasin B. The oocytes were enucleated as described in our previous publication [21]. Oocytes were stained with 1 μg/mL Hoechst 33,342, and using microinjection needle, the first polar body was aspirated along with approximately 15% of the adjacent cytoplasm under UV light irradiation. For subsequent SCNT, the absolutely enucleated oocytes were used. A single donor cell with a round and smooth surface was microinjected into the perivitelline space of each enucleated oocyte. The reconstructed oocytes were washed thrice in porcine zygote medium 3 (PZM-3) medium [23], and then electrically fused using two direct current pulses at 150 V/mm for 50 μs, and subsequently incubated in PZM-3 medium for 1 h at 38.5 °C in a humidified atmosphere of 5% CO_2_. The reconstructed oocytes were activated by two direct current pulses of 100 V/mm for 20 μs, and incubated in PZM-3 medium supplemented with 5 μg/mL cytochalasin B for 4 h. The activated embryos were finally cultured in fresh PZM-3 medium for 20 h at 38.5 °C in a humidified incubator with 5% CO_2_ and used for embryo transfer.

The embryo transfer was conducted as previously reported [17], but with some adjustments. In brief, SCNT embryos cultured for 20 h in vitro were examined and the abnormal embryos were removed. Only embryos with intact cytoplasm or normal cleavage were employed for embryo transfer. These normal SCNT embryos were loaded into a transparent transfer tube and placed in a portable incubator before transferring into surrogate sow. Estrus-synchronized Landrace and Yorkshire sows in parity 2–5 and showing natural standing estrus within 30–50 h were used as embryo recipients. These sows were anesthetized with ketamine (25 mg/kg body weight) and xylazine (1.1 mg/kg body weight) for induction and 3% isoflurane for maintenance. A single oviduct was exposed through surgery. The SCNT embryos with 100 μL of culture medium were placed directly using a syringe into the oviduct of the sow. One month after embryo transfer, the pregnancy status of the surrogate sows was monitored by ultrasound equipped with a convex transducer. If spontaneous farrowing did not occur until gestation day 116, the surrogate sows were injected with a prostaglandin analog (cloprostenol, 200 μg/surrogates); after about 24 h, they delivered vaginally under supervision or with assistance. The total number of newborn cloned piglets (including live-born and stillborn) in each litter was recorded to calculate the average litter size of farrowed surrogates. To ensure uniformity, all embryo preparation and transfer procedures were conducted by a single, dedicated team according to a uniform, established protocol.

### 2.6. Statistical Analysis

Statistical analyses were performed based on the structure of the outcome variables. Pregnancy rate, delivery rate, and overall cloning efficiency were presented as percentages. Comparisons of these rates between groups were performed using Pearson’s chi-square test. When the overall chi-square test was significant (*p* < 0.05), post hoc pairwise comparisons were conducted with Bonferroni correction to adjust for multiple comparisons. Comparisons of means between two independent groups were performed using Student’s *t*-test. All statistical analyses were performed using R software (version 4.5.1).

## 3. Results

### 3.1. Overview of Five-Year Large-Scale Cloning Production

Over the course of this large-scale production study, a total of 367,701 porcine SCNT embryos were transferred into 2019 surrogate sows in our laboratory from 2021 to 2025. Per surrogate, the average number of SCNT embryos transferred was 182 (range: 112–300). A total of 1488 surrogates (73.70%) became pregnant and 1316 surrogates (88.44%) delivered 7781 piglets, with an average cloning efficiency of 2.12% (Table 1). Donor cells were obtained from adult Duroc, Large White, Pietrain, Yorkshire, and GM pigs.

### 3.2. Effect of Embryo Transfer Season on Cloning Outcomes

The season in which surrogates received embryo transfer significantly influenced pregnancy maintenance, successful delivery, the number of piglets per litter, and the overall cloning efficiency. Regarding pregnancy rates, those for surrogates receiving embryo transfers in Summer were significantly lower than that those in Spring (67.90% vs. 76.62%, *p* = 0.0462), but did not differ significantly from Spring, Autumn (72.83%), and Winter (72.43%). A similar pattern was observed for the delivery rates. The delivery rate in Summer was significantly lower than in Spring (80.00% vs. 89.67%, *p* < 0.01), Autumn (80.00% vs. 89.39%, *p* = 0.044), and Winter (80.00% vs. 89.28%, *p* = 0.0346), and no significant differences were found among the latter three seasons. In addition, the overall cloning efficiency was significantly compromised in Summer compared to the other three seasons. The efficiencies in Spring (1.74% vs. 2.20%, *p* < 0.01), Autumn (1.74% vs. 2.05%, *p* < 0.01), and Winter (1.74% vs. 2.22%, *p* < 0.01) were statistically similar and markedly higher. Regarding the average litter size, Winter yielded the highest numerical value (6.23 ± 2.50), which was significantly greater than that in both Spring (5.76 ± 2.45) and Autumn (5.76 ± 2.46). The average litter size in Summer (6.11 ± 2.93) was not statistically different from that in any other season. Collectively, Summer conditions adversely affected cloning success by reducing pregnancy establishment, lowering maintenance, and diminishing the overall cloning efficiency. Outcomes in Spring, Autumn, and Winter were more favorable and generally comparable.

### 3.3. Influence of Donor Cell Breed on Cloning Efficiency

The breed of the donor cell was a significant factor affecting cloning outcomes, and this influence was consistently observed in both wild-type (WT) and genetically modified (GM) cell populations, which were analyzed separately (Table 2 and Table 3).

In the analysis of WT-donor cells, pregnancy rates showed no significant differences among breeds, ranging from 72.00% to 75.33%. Similarly, delivery rates showed no significant differences, despite numerical variations from 72.22% to 90.90% (Table 2). However, both the number of piglets per litter and cloning efficiency exhibited marked breed-specific variations. WT-Pietrain cells yielded a markedly higher number of piglets per litter (9.77 ± 3.09) and achieved the highest cloning efficiency (2.48%). This litter size was dramatically higher than that of WT-Duroc (9.77 ± 3.09 vs. 5.90 ± 2.49, *p* < 0.01), WT-Large White (9.77 ± 3.09 vs. 5.51 ± 2.29, *p* < 0.01), and WT-Yorkshire (9.77 ± 3.09 vs. 6.17 ± 2.73, *p* < 0.01) cells. WT-Duroc, WT-Large White, and WT-Yorkshire did not differ significantly from each other in litter size. In terms of cloning efficiency, WT-Pietrain achieved the highest efficiency (2.48%), and no significant difference was observed between WT-Pietrain and WT-Duroc (2.23%). Moreover, the cloning efficiency of WT-Duroc was significantly higher than both WT-Large White (2.23% vs. 1.95%, *p* < 0.001) and WT-Yorkshire (2.23% vs. 1.81%, *p* < 0.001). Similarly, WT-Pietrain showed significantly higher efficiency than both WT-Large White (2.48% vs. 1.81%, *p* < 0.001) and WT-Yorkshire (2.48% vs. 1.95%, *p* = 0.00341). No significant difference was observed between WT-Large White and WT-Yorkshire.

A similar pattern was observed with genetically modified (GM) donor cells, further supporting the significant impact of cell breed on cloning outcomes. Analysis revealed significant variations in cloning efficiency among GM breeds, while pregnancy rates, delivery rates, and the number of piglets per litter remained comparable (Table 3). Among the GM-donor cell breeds, pregnancy rates were highest with GM-Duroc (74.65%) and lowest with GM-Large White (63.64%), though these differences were not statistically significant. Similarly, for delivery rates, the highest value was observed with GM-Large White (92.86%) and the lowest with GM-Bama (74.42%), with no significant differences across groups. The litter size was largest for GM-Bama and smallest for GM-Large White (6.84 ± 2.93 vs. 5.31 ± 2.36, *p* = 0.745), but the difference was not statistically significant. However, cloning efficiency varied significantly among GM breeds (Table 3). GM-Duroc achieved the highest efficiency (2.40%), and no significant difference was observed between GM-Duroc and GM-Yorkshire (2.15%). The cloning efficiency of GM-Duroc was significantly higher than both GM-Large White (2.40% vs. 1.68%, *p* = 0.0492) and GM-Bama (2.40% vs. 1.73%, *p* = 0.00160). Additionally, GM-Yorkshire showed significantly higher efficiency than GM-Bama (2.15% vs. 1.73%, *p* = 0.0339).

These results indicated that donor cell breeds significantly affected cloning efficiency in analyses of both WT and GM donor cells.

### 3.4. Comparison of Cloning Efficiency Between WT and GM Donor Cells

**Figure 1:**
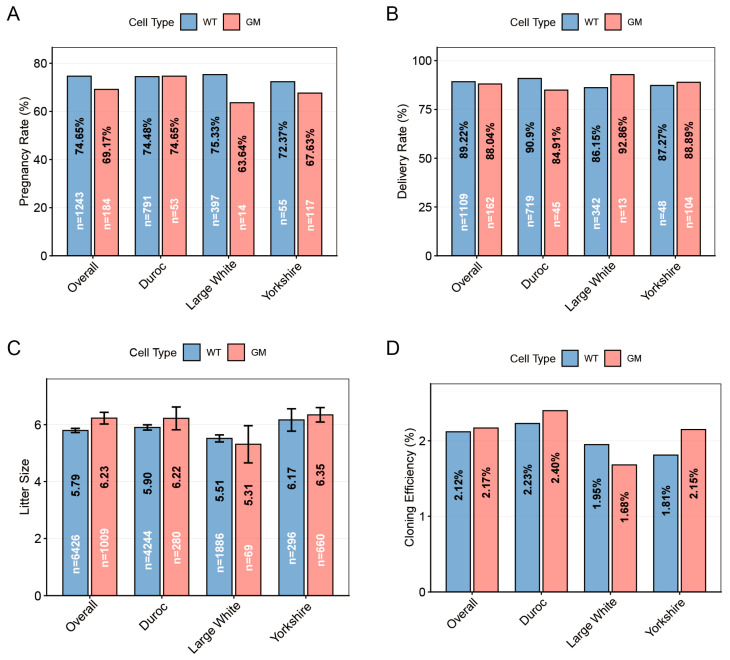
Comparison of cloning efficiency between WT and GM donor cells in porcine somatic cell nuclear transfer. Data are presented for the overall population (representing the combined data from all three breeds) and separately for three pig breeds (Duroc, Large White, and Yorkshire). Pregnancy rate (**A**), delivery rate (**B**), average litter size (**C**) and cloning efficiency (**D**) of surrogate sows stratified by donor cell types.

To evaluate the effect of genetic modification on the cloning efficiency of pigs, we compared the developmental outcomes of SCNT embryos using WT and GM donor cells across three pig breeds: Duroc, Large White, and Yorkshire. As shown as Figure 1, no statistically significant differences were observed between the WT and GM groups in terms of the pregnancy rate, delivery rate, average litter size, or cloning efficiency. Notably, however, in the Large White breed, the GM group showed a consistent downward trend in both the pregnancy rate (75.33% vs. 63.64%, *p* = 0.323; Figure 1A) and cloning efficiency (1.95% vs. 1.68%, *p* = 0.237; Figure 1D) compared to the WT group, although these differences were not statistically significant.

To control for interannual variability and enable a direct comparison, we analyzed the production outcomes of WT and GM donor cells within each calendar year from 2022 to 2025 (Table S1). Embryos derived from each donor type were transferred into recipient sows, and key reproductive parameters were evaluated on an annual basis. Pregnancy rates in recipients receiving WT-derived embryos ranged from 70.69% to 93.53%, while those receiving GM-derived embryos ranged from 64.10% to 92.31%. No statistically significant differences between donor cell types were observed in any individual year (all *p* > 0.05). Similarly, delivery rates remained high and comparable between groups throughout the study period. The total cloning efficiency showed a consistent upward trend from 2022 to 2025 for both WT (from 1.72% to 2.41%) and GM (from 1.87% to 3.04%) donor cells. The average litter size was similar between groups in most years, with a statistically significant difference observed only in 2024, where GM donor cells yielded a higher average litter size than WT (6.11 ± 2.47 vs. 5.47 ± 2.29 piglets, *p* = 0.031). In other years, litter sizes did not differ significantly between donor types. Collectively, these within-year comparisons indicate that genetic modification of donor cells did not adversely affect annual cloning efficiency or most reproductive outcomes in recipients.

### 3.5. Transferred Embryo Number Significantly Affects Cloning Efficiency

According to previous reports, the production efficiency of cloned pigs is influenced by the number of SCNT embryos transferred. For this analysis, we categorized the number of transferred embryos into four groups: 100–150 embryos, 151–200 embryos, 201–250 embryos, and 251–300 embryos. As shown in Table 4, the number of embryos transferred per surrogate had minimal impact on pregnancy rates, delivery rates, and average litter size across the different transfer groups. Statistical analysis confirmed no significant differences in pregnancy rates, delivery rates, and average litter size across the transferred embryo number groups. However, a marked and statistically significant inverse relationship was observed between the transferred embryo number into surrogates and cloning efficiency (*p* < 0.01). Specifically, the cloning efficiency achieved with 100–150 transferred embryos was significantly higher than that of the 151–200 transferred-embryo group (2.88% vs. 1.91%, *p* < 0.01), the 201–250 transferred-embryo group (2.88% vs. 1.80%, *p* < 0.01), and the 251–300 transferred-embryo group (2.88% vs. 1.53%, *p* < 0.01). Furthermore, the cloning efficiency of the 151–200 transferred-embryo group was significantly higher than that of both the 201–250 (1.91% vs. 1.80%, *p* < 0.01) and 251–300 transferred-embryo groups (1.91% vs. 1.53%, *p* < 0.01), while no significant difference was observed between the 201–250 and 251–300 transferred-embryo groups (*p* = 0.165).

To further verify the effects of the number of transferred embryos on the production efficiency of cloned pigs, we randomly transferred approximately 150 or 200 cloned embryos generated from the same donor cells and constructed on the same day to surrogate sows, respectively. This key finding was robustly validated by the controlled experiment, presented in Table 5. Although increasing the number of transferred embryos from 150 to 200 showed a trend toward reduced pregnancy rates (86.67% vs. 60.00%, *p* = 0.0986), this difference did not reach statistical significance. Recipients receiving 150 embryos exhibited significantly larger average litter sizes (6.38 ± 3.04 vs. 4.22 ± 1.39, *p* = 0.0377), and a nearly threefold greater cloning efficiency (3.66% vs. 1.27%, *p* < 0.01) compared to those receiving 200 embryos.

These results demonstrate that the number of embryos transferred to surrogates significantly affected the efficiency of cloned pig production. Specifically, transferring 150 embryos yielded optimal outcomes, with significantly higher cloning efficiency and larger litter sizes compared to 200 embryo transfers, while maintaining comparable pregnancy and delivery rates. This finding provides important guidance for optimizing SCNT protocols in pig cloning.

## 4. Discussion

SCNT technology is currently the most efficient and precise approach to generate genetically engineered pig models for agricultural applications and biomedical research [1,2,3], yet its overall cloning efficiency is constrained by multiple interacting factors. In this study, we reported that the season of transferred embryos, breeds of donor cells, and number of embryos transferred can affect the production of cloned pigs. These findings have immediate implications for enhancing the scale, reproducibility, and economic viability of pig cloning in both biomedical research and agricultural applications.

In this study, surrogate sows receiving embryo transfers during Summer exhibited the lowest pregnancy, delivery rate, and cloning efficiency rates compared to the other seasons. In contrast, the performance metrics for Spring, Autumn, and Winter transfers were largely comparable, with Spring showing only a slight, non-significant advantage. The significantly compromised pregnancy and delivery rates in surrogate sows receiving embryo transfers in Summer indicate a substantive failure in the establishment and maintenance of early pregnancy. Environmental heat stress can impair oocyte quality [24,25], which adversely affects the subsequent developmental potential of the SCNT embryos. Furthermore, in both gilts and sows, sustained heat stress and a long photoperiod during Summer are critical factors that not only suppress feed intake but also disrupt the imbalance of the hypothalamic–hypophysial–ovarian axis [26,27], thereby adversely affecting pregnancy maintenance. These impairments may therefore underlie the substantially lower cloning efficiency in surrogate sows that received embryo transfer in Summer. Although previous studies have established that the season of embryo transfer is an important factor for cloning efficiency in surrogate sows, not all studies have reported Summer as the most detrimental period [10,28,29]. These discrepancies are likely attributable to geographic variations in specific climatic conditions.

Donor cells are a critical determinant of cloning efficiency, with previous studies demonstrating the importance of donor cell characteristics, including size [13], source [29,30], type [31] and other factors [32,33]. Among WT donor cells, Pietrain and Duroc donor cell-derived SCNT embryos yielded the highest total cloning efficiency, while Large White and Yorkshire breeds showed lower performance. This trend persisted with GM donor cells, where GM-Duroc and GM-Yorkshire demonstrated better efficiency than GM-Large White and GM-Bama. These findings collectively suggest that specific pig breeds possess breed-specific characteristics that facilitate more effective nuclear reprogramming. Furthermore, we specifically investigated the impact of GM donor cells on cloning outcomes. Our large-scale data clearly indicate that the use of GM donor cells does not exert a significant negative impact on the efficiency of pig cloning compared to their wild-type counterparts. These findings collectively indicate that the genetic background of donor cells from different breeds directly impact the outcome of nuclear reprogramming during SCNT.

The number of embryos transferred is widely recognized as a key factor influencing the outcomes of pig cloning. In 2013, our team identified the transfer of 200–249 embryos per recipient as an optimal number range [31]. However, after more than a decade of large-scale data tracking, our current investigation refines this understanding. Our current analysis reveals that increasing the number of embryos transferred from 100–150 to 251–300 had no significant impact on the pregnancy rate, delivery rate, or average litter size of surrogate sows, but was negatively correlated with the overall cloning efficiency. This relationship is definitively supported by our controlled experiment demonstrating superior cloning efficiency and higher litter size in recipients receiving 150 versus 200 embryo transfers. These findings demonstrate that exceeding an optimal embryo number does not improve cloning outcomes, despite the established minimum requirement of four embryos for pregnancy maintenance in pigs. Notably, compared with data from a decade ago, pregnancy rates in the 200–299 embryo transfer groups have risen to exceed 70% (from below 60%), and cloning efficiency showed a >4-fold increase, indicating substantial improvements in SCNT embryo quality. However, excessive embryo development leads to mutual exclusion because the uterine capacity in sows limits the number of fetuses developing to term, resulting in the loss of supernumerary fetuses [34,35]. Thus, with the improved quality of individual cloned embryos, transferring excessive numbers becomes counterproductive for embryonic development to term. Our findings establish that surrogates receiving 100–150 cloned embryo transfers maintain comparable pregnancy rates, delivery rates, and average litter sizes while achieving significantly higher cloning efficiency than those receiving larger numbers of embryos.

This study retrospectively analyzed the embryo transfer records of our team rather than prospective new experiments. Thus, the experimental conditions, such as surrogate sows (including age, weight, parity, and breeds), passages of donor cell lines, the embryo transfer operation, the number of embryos transferred, and season, were not tightly controlled and their variations may have influenced the outcomes. Additionally, our data cannot determine whether transferring fewer than 100 embryos would lead to improved cloning outcomes in surrogate sows, which warrants further investigation. Furthermore, the lack of systematically recorded environmental data precludes a direct correlation between climatic conditions and the observed seasonal variations in cloning efficiency. Future studies integrating real-time environmental monitoring are warranted to address this. Moreover, our statistical approach, employing direct group comparisons, did not account for the simultaneous effects of multiple variables; thus, it cannot distinguish their individual and interactive influences on the outcomes. However, the total number of embryos transferred (367,701) and the number of surrogate sows (2019) were large, and data collected were for a longer duration. Therefore, this study provides valuable information for further research on porcine cloning.

## 5. Conclusions

Our large-scale retrospective analysis demonstrates that the genetic background of donor cells significantly influences the efficiency of pig cloning. SCNT embryos derived from Duroc and Pietrain donor cells yielded significantly higher production efficiency compared to those from Large White and Yorkshire breeds. Furthermore, our findings indicate that Spring is the optimal season for cloned pig production in South China, and transferring 100–150 embryos per surrogate sow represents the most efficient strategy to maximize overall cloning outcomes. These insights provide valuable guidance for optimizing large-scale SCNT protocols in swine.

## Figures and Tables

**Table 1:** Cloning efficiency statistics and seasonal variations in a large-scale production of cloned pigs.

Season	No. Surrogates (Embryos Transferred)	No. Pregnancy (%)	No. Delivery (%)	No. Piglets (Mean ± SD)	Total Cloning Efficiency (%)
Spring	834 (149,838)	639 (76.62) ^a^	573 (89.67) ^a^	3301 (5.76 ± 2.45 ^a^)	2.20 ^a^
Summer	243 (46,333)	165 (67.90) ^b^	132 (80.00) ^b^	807 (6.11 ± 2.93 ^ab^)	1.74 ^b^
Autumn	427 (77,956)	311 (72.83) ^ab^	278 (89.39) ^a^	1600 (5.76 ± 2.46 ^a^)	2.05 ^a^
Winter	515 (93,574)	373 (72.43) ^ab^	333 (89.28) ^a^	2073 (6.23 ± 2.50 ^b^)	2.22 ^a^
Total	2019 (367,701)	1488 (73.70)	1316 (88.44)	7781 (5.91 ± 2.52)	2.12

Pregnancy rate = pregnant recipient sows/number of recipient sows. Delivery rate = delivery sows/pregnant sows. Average litter size = total litter size/number of delivery sows. Total cloning efficiency = litter size/total number of embryos transferred. Values in the same column labeled with different superscripts differ at *p* < 0.05.

**Table 2:** Cloning outcomes using wild-type donor cells from different pig breeds (WT).

Donor Cell Breeds	No. Surrogates (Embryos Transferred)	No. Pregnancy (%)	No. Delivery (%)	No. Piglets (Mean ± SD)	Total Cloning Efficiency (%)
WT-Duroc	1062 (190,401)	791 (74.48)	719 (90.90)	4244 (5.90 ± 2.49 ^a^)	2.23 ^a^
WT-Large White	527 (96,644)	397 (75.33)	342 (86.15)	1886 (5.51 ± 2.29 ^b^)	1.95 ^b^
WT-Yorkshire	76 (16,340)	55 (72.37)	48 (87.27)	296 (6.17 ± 2.73 ^ab^)	1.81 ^b^
WT-Pietrain	25 (5130)	18 (72.00)	13 (72.22)	127 (9.77 ± 3.09 ^c^)	2.48 ^a^
WT total	1690 (308,515)	1261 (74.65)	1122 (88.98)	6553 (5.84 ± 2.49)	2.12

Average litter size = total litter size/number of delivery sows. Total cloning efficiency = litter size/total number of embryos transferred. WT: wild type. Values in the same column labeled with different superscripts differ at *p* < 0.05.

**Table 3:** Cloning outcomes using genetically modified donor cells from different pig breeds (GM).

Donor Cell Breeds	No. Surrogates (Embryos Transferred)	No. Pregnancy (%)	No. Delivery (%)	No. Piglets (Mean ± SD)	Total Cloning Efficiency (%)
GM-Duroc	71 (11,663)	53 (74.65)	45 (84.91)	280 (6.22 ± 2.70)	2.40 ^a^
GM-Large White	22 (4110)	14 (63.64)	13 (92.86)	69 (5.31 ± 2.36)	1.68 ^b^
GM-Yorkshire	173 (30,749)	117 (67.63)	104 (88.89)	660 (6.35 ± 2.61)	2.15 ^a^
GM-Bama	63 (12,664)	43 (68.25)	32 (74.42)	219 (6.84 ± 2.93)	1.73 ^bc^
GM total	329 (59,186)	227 (69.00)	194 (85.46)	1228 (6.33 ± 2.67)	2.08

Average litter size = total litter size/number of delivery sows. Total cloning efficiency = litter size/total number of embryos transferred. GM: genetic modification. Values in the same column labeled with different superscripts differ at *p* < 0.05.

**Table 4:** Cloning efficiency following transfer of different numbers of cloned pig embryos.

No. Transferred Embryos per Surrogates	No. Surrogates (Embryos Transferred)	No. Pregnancy (%)	No. Delivery (%)	No. Piglets (Mean ± SD)	Total Cloning Efficiency (%)
100~150	340 (46,421)	263 (77.35)	234 (88.97)	1337 (5.71 ± 2.40)	2.88 ^a^
151~200	1120 (196,465)	822 (73.39)	719 (87.47)	4206 (5.85 ± 2.46)	1.91 ^b^
201~250	512 (113,649)	365 (71.29)	328 (89.86)	2040 (5.85 ± 2.47)	1.80 ^c^
251~300	47 (12,977)	38 (80.85)	35 (92.10)	198 (5.66 ± 2.84)	1.53 ^c^

Average litter size = total litter size/number of delivery sows. Total cloning efficiency = litter size/total number of embryos transferred. Values in the same column labeled with different superscripts differ at *p* < 0.05.

**Table 5:** Comparison of cloning efficiency following transfer of 150 versus 200 embryos per surrogate.

No. Transferred Embryos per Surrogates	No. Surrogates (Embryos Transferred)	No. Pregnancy (%)	No. Delivery (%)	No. Piglets (Mean ± SD)	Total Cloning Efficiency (%)
150	15 (2265)	13 (86.67)	13 (100.00)	83 (6.38 ± 3.04 ^a^)	3.66 ^a^
200	15 (2985)	9 (60.00)	9 (100.00)	38 (4.22 ± 1.39 ^b^)	1.27 ^b^

Average litter size = total litter size/number of delivery sows. Total cloning efficiency = litter size/total number of embryos transferred. Values in the same column labeled with different superscripts differ at *p* < 0.05.

## Data Availability

All data generated or analyzed during this study are included in this published article. Additional datasets are available from the corresponding authors on reasonable request.

## Supplementary Materials

The following supporting information can be downloaded at: https://www.mdpi.com/article/10.3390/ani16020168/s1, Table S1: Comparison of annual production outcomes between wild-type and genetically modified donor cells in porcine somatic cell nuclear transfer.

## Abbreviations

The following abbreviations are used in this manuscript:

SCNT	Somatic cell nuclear transfer
GM	Genetically modified
WT	Wild type

## References

[1] Galli C., Lazzari G. (2021). 25th ANNIVERSARY OF CLONING BY SOMATIC-CELL NUCLEAR TRANSFER: Current applications of SCNT in advanced breeding and genome editing in livestock. Reproduction.

[2] Dai Y., Vaught T.D., Boone J., Chen S.H., Phelps C.J., Ball S., Monahan J.A., Jobst P.M., McCreath K.J., Lamborn A.E. (2002). Targeted disruption of the alpha1,3-galactosyltransferase gene in cloned pigs. Nat. Biotechnol..

[3] Yan S., Tu Z.C., Liu Z.M., Fan N.N., Yang H.M., Yang S., Yang W.L., Zhao Y., Ouyang Z., Lai C.D. (2018). A Huntingtin Knockin Pig Model Recapitulates Features of Selective Neurodegeneration in Huntington’s Disease. Cell.

[4] Niemann H., Lucas-Hahn A. (2012). Somatic cell nuclear transfer cloning: Practical applications and current legislation. Reprod. Domest. Anim..

[5] Park J.-K., Lee Y.-K., Lee P., Chung H.-J., Kim S., Lee H.-G., Seo M.-K., Han J.-H., Park C.-G., Kim H.-T. (2006). Recombinant human erythropoietin produced in milk of transgenic pigs. J. Biotechnol..

[6] Onishi A., Iwamoto M., Akita T., Mikawa S., Takeda K., Awata T., Hanada H., Perry A.C.F. (2000). Pig cloning by microinjection of fetal fibroblast nuclei. Science.

[7] Vajta G., Gjerris M. (2006). Science and technology of farm animal cloning: State of the art. Anim. Reprod. Sci..

[8] Liu T., Dou H., Xiang X., Li L., Li Y., Lin L., Pang X., Zhang Y., Chen Y., Luan J. (2015). Factors Determining the Efficiency of Porcine Somatic Cell Nuclear Transfer: Data Analysis with Over 200,000 Reconstructed Embryos. Cell. Reprogram..

[9] Liu Y., Li J., Løvendahl P., Schmidt M., Larsen K., Callesen H. (2015). In vitro manipulation techniques of porcine embryos: A meta-analysis related to transfers, pregnancies and piglets. Reprod. Fertil. Dev..

[10] Koo O.J., Kang J.T., Kwon D.K., Park H.J., Lee B.C. (2010). Influence of ovulation status, seasonality and embryo transfer method on development of cloned porcine embryos. Reprod. Domest. Anim..

[11] Zhao H., Dong Y., Zhang Y., Wu X., Zhang X., Liang Y., Li Y., Zeng F., Shi J., Zhou R. (2022). Supplementation of SDF1 during Pig Oocyte In Vitro Maturation Improves Subsequent Embryo Development. Molecules.

[12] Jin J.X., Lee S., Setyawan E.M.N., Taweechaipaisankul A., Kim G.A., Han H.J., Ahn C., Lee B.C. (2018). A potential role of knockout serum replacement as a porcine follicular fluid substitute for in vitro maturation: Lipid metabolism approach. J. Cell. Physiol..

[13] Jiao D., Cheng W., Zhang X., Zhang Y., Guo J., Li Z., Shi D., Xiong Z., Qing Y., Jamal M.A. (2021). Improving porcine SCNT efficiency by selecting donor cells size. Cell Cycle.

[14] Hyun H., Lee S.E., Son Y.J., Shin M.Y., Park Y.G., Kim E.Y., Park S.P. (2016). Cell Synchronization by Rapamycin Improves the Developmental Competence of Porcine SCNT Embryos. Cell. Reprogram..

[15] Kurome M., Fujimura T., Murakami H., Takahagi Y., Wako N., Ochiai T., Miyazaki K., Nagashima H. (2003). Comparison of electro-fusion and intracytoplasmic nuclear injection methods in pig cloning. Cloning Stem Cells.

[16] Lee K., Davis A., Zhang L., Ryu J., Spate L.D., Park K.-W., Samuel M.S., Walters E.M., Murphy C.N., Machaty Z. (2015). Pig oocyte activation using a Zn(2)(+) chelator, TPEN. Theriogenology.

[17] Shi J., Zhou R., Luo L., Mai R., Zeng H., He X., Liu D., Zeng F., Cai G., Ji H. (2015). Influence of embryo handling and transfer method on pig cloning efficiency. Anim. Reprod. Sci..

[18] Huang Y., Ouyang H., Yu H., Lai L., Pang D., Li Z. (2013). Efficiency of porcine somatic cell nuclear transfer—A retrospective study of factors related to embryo recipient and embryos transferred. Biol. Open.

[19] Polge C., Rowson L.E.A., Chang M.C. (1966). The effect of reducing the number of embryos during early stages of gestation on the maintenance of pregnancy in the pig. J. Reprod. Fertil..

[20] Zhao H., He X., Zhang X., Shi J., Zhou R., Mai R., Su Q., Cai G., Huang S., Xu Z. (2023). Progesterone and Androstenedione Are Important Follicular Fluid Factors Regulating Porcine Oocyte Maturation Quality. Animals.

[21] Zhao H., Xie S., Zhang N., Ao Z., Wu X., Yang L., Shi J., Mai R., Zheng E., Cai G. (2020). Source and Follicular Fluid Treatment During the In Vitro Maturation of Recipient Oocytes Affects the Development of Cloned Pig Embryo. Cell. Reprogram..

[22] Zhang X., Li Z., Yang H., Liu D., Cai G., Li G., Mo J., Wang D., Zhong C., Wang H. (2018). Novel transgenic pigs with enhanced growth and reduced environmental impact. eLife.

[23] Yoshioka K., Suzuki C., Itoh S., Kikuchi K., Iwamura S., Rodriguez-Martinez H. (2003). Production of piglets derived from in vitro-produced blastocysts fertilized and cultured in chemically defined media: Effects of theophylline, adenosine, and cysteine during in vitro fertilization. Biol. Reprod..

[24] Hale B.J., Hager C.L., Seibert J.T., Selsby J.T., Baumgard L.H., Keating A.F., Ross J.W. (2017). Heat stress induces autophagy in pig ovaries during follicular development. Biol. Reprod..

[25] Yin C., Liu J., He B., Jia L., Gong Y., Guo H., Zhao R. (2019). Heat stress induces distinct responses in porcine cumulus cells and oocytes associated with disrupted gap junction and trans-zonal projection colocalization. J. Cell. Physiol..

[26] De Rensis F., Ziecik A.J., Kirkwood R.N. (2017). Seasonal infertility in gilts and sows: Aetiology, clinical implications and treatments. Theriogenology.

[27] Bertoldo M.J., Holyoake P.K., Evans G., Grupen C.G. (2012). Seasonal variation in the ovarian function of sows. Reprod. Fertil. Dev..

[28] Huang T., Li Z., Lv P., Zhou J., Ye C., Li A., Yuan M., Liu H., Cao G. (2023). Influence of season and conditions of surrogate sows on efficiency of somatic cell cloning production. Reprod. Domest. Anim..

[29] Kurome M., Geistlinger L., Kessler B., Zakhartchenko V., Klymiuk N., Wuensch A., Richter A., Baehr A., Kraehe K., Burkhardt K. (2013). Factors influencing the efficiency of generating genetically engineered pigs by nuclear transfer: Multi-factorial analysis of a large data set. BMC Biotechnol..

[30] Hua Z., Xu G., Liu X., Bi Y., Xiao H., Hua W., Li L., Zhang L., Ren H., Zheng X. (2016). Impact of different sources of donor cells upon the nuclear transfer efficiency in Chinese indigenous Meishan pig. Pol. J. Vet. Sci..

[31] Li Z., Shi J., Liu D., Zhou R., Zeng H., Zhou X., Mai R., Zeng S., Luo L., Yu W. (2013). Effects of donor fibroblast cell type and transferred cloned embryo number on the efficiency of pig cloning. Cell. Reprogram..

[32] Yoo J.-G., Kim B.-W., Park M.-R., Kwon D.-N., Choi Y.-J., Shin T.-S., Cho B.-W., Seo J., Kim J.-H., Cho S.-K. (2017). Influences of somatic donor cell sex on in vitro and in vivo embryo development following somatic cell nuclear transfer in pigs. Asian-Australas. J. Anim. Sci..

[33] Cho J., Kim G., Qamar A.Y., Fang X., Roy P.K., Tanga B.M., Bang S., Kim J.K., Galli C., Perota A. (2022). Improved efficiencies in the generation of multigene-modified pigs by recloning and using sows as the recipient. Zygote.

[34] Christenson R.K., Leymaster K.A., Young L.D. (1987). Justification of unilateral hysterectomy-ovariectomy as a model to evaluate uterine capacity in swine. J. Anim. Sci..

[35] Bennett G.L., Leymaster K.A. (1989). Integration of ovulation rate, potential embryonic viability and uterine capacity into a model of litter size in swine. J. Anim. Sci..

