# Supplementary material for: Factors Influencing the Production Efficiency of Cloned Pigs: A Large-Scale Retrospective Analysis
Source: Animals (Basel). 2026 Jan 7;16(2):168. doi: 10.3390/ani16020168 (PMC12837946; doi:10.3390/ani16020168)
Supplement: Supplementary file 1 [file animals-16-00168-s001.zip › animals-4034178-supplementary.pdf]

Table S1. Comparison of annual production outcomes between wild-type and genetically modified donor cells in porcine somatic cell nuclear transfer.

Year	Genetic types	No. surrogates (embryos transferred)	No. pregnancy (%)	No. delivery (%)	No. piglets (mean± SD)	Total cloning efficiency (%)
2022	WT	331(69,500)	234(70.69)	207(88.46)	1,192(5.76±2.57)	1.72
	GM	78(15,737)	50(64.10)	44(88.00)	294(6.68±2.91)	1.87
2023	WT	311(61,434)	239(76.85)	222(92.89)	1,325(5.97±6.12)	2.16
	GM	36(6,350)	26(72.22)	24(92.31)	168(6.12±2.54)	2.18
2024	WT	628(106,438)	477(75.96)	420(88.05)	2,296(5.47±2.29[a])	2.16
	GM	139(22,229)	96(69.06)	82(85.42)	501(6.11±2.47[b])	2.25
2025	WT	309(64,902)	289(93.53)	255(88.24)	1,562(6.13±2.37)	2.41
	GM	13(2,206)	12(92.31)	12(100)	67(5.58±2.64)	3.04

Average litter size = total litter size/number of delivery sows; Total cloning efficiency = litter size/total number of embryos transferred. Values in the same column labeled with different superscripts differ at $P<0.05$.

Year 2022: Pregnancy rate: $P = 0.2556$; Delivery rate: $P = 0.857$; Average litter size: $P = 0.056$; Cloning efficiency: $P = 0.1851$.

Year 2023: Pregnancy rate: $P = 0.3082$; Delivery rate: $P = 0.476$; Average litter size: $P = 0.777$; Cloning efficiency: $P = 0.8956$.

Year 2024: Pregnancy rate: $P = 0.0908$; Delivery rate: $P = 0.475$; Average litter size: $P = 0.031$; Cloning efficiency: $P = 0.3686$.

Year 2025: Pregnancy rate: $P = 0.5910$; Delivery rate: $P = 0.373$; Average litter size: $P = 0.499$; Cloning efficiency: $P = 0.0585$.
